# Pro-Inflammatory versus Immunomodulatory Effects of Silver Nanoparticles in the Lung: The Critical Role of Dose, Size and Surface Modification

**DOI:** 10.3390/nano7100300

**Published:** 2017-09-29

**Authors:** Francesca Alessandrini, Antje Vennemann, Silvia Gschwendtner, Avidan U. Neumann, Michael Rothballer, Tanja Seher, Maria Wimmer, Susanne Kublik, Claudia Traidl-Hoffmann, Michael Schloter, Martin Wiemann, Carsten B. Schmidt-Weber

**Affiliations:** 1 Center of Allergy and Environment (ZAUM), Technical University and Helmholtz Center Munich, Member of the German Center for Lung Research (DZL), Ingolstädter Landstr. 1, 85764 Neuherberg, Germany; tanja.seher@helmholtz-muenchen.de (T.S.); maria.wimmer84@web.de (M.W.); csweber@tum.de (C.B.S.-W.); 2 IBE R & D Institute for Lung Health gGmbH, Mendelstr. 11, 48149 Münster, Germany; vennemann@ibe-ms.de (A.V.); martin.wiemann@ibe-ms.de (M.W.); 3 Research Unit for Comparative Microbiome Analysis, Helmholtz Center Munich, Ingolstädter Landstr. 1, 85764 Neuherberg, Germany; silvia.gschwendtner@helmholtz-muenchen.de (S.G.); susanne.kublik@helmholtz-muenchen.de (S.K.); schloter@helmholtz-muenchen.de (M.S.); 4 Chair and Institute of Environmental Medicine, UNIKA-T, Technical University of Munich and Helmholtz Center Munich, ,Neusäßer Str. 17, 86156 Augsburg, Germany; 5 CK-CARE, Christine Kühne-Center for Allergy and Research and Education, Herman-Burchard-Strasse 1, 7265 Davos Wolfgang, Switzerland; auneumann@gmail.com (A.U.N.); claudia.traidl-hoffmann@tum.de (C.T.-H.); 6 Institute of Network Biology (INET), Helmholtz Center Munich, Ingolstädter Landstr. 1, 85764 Neuherberg, Germany; rothballer@helmholtz-muenchen.de

**Keywords:** silver nanoparticles, adjuvant, allergic inflammation, surface modification

## Abstract

The growing use of silver nanoparticles (Ag-NPs) in consumer products raises concerns about their toxicological potential. The purpose of the study was to investigate the size- and coating-dependent pulmonary toxicity of Ag-NPs in vitro and in vivo, using an ovalbumin (OVA)-mouse allergy model. Supernatants from (5.6–45 µg/mL) Ag50-PVP, Ag200-PVP or Ag50-citrate-treated NR8383 alveolar macrophages were tested for lactate dehydrogenase and glucuronidase activity, tumor necrosis factor (TNF)-α release and reactive oxygen species (ROS) production. For the in vivo study, NPs were intratracheally instilled in non-sensitized (NS) and OVA-sensitized (S) mice (1–50 µg/mouse) prior to OVA-challenge and bronchoalveolar lavage fluid (BALF) inflammatory infiltrate was evaluated five days after challenge. In vitro results showed a dose-dependent cytotoxicity of Ag-NPs, which was highest for Ag50-polyvinilpyrrolidone (PVP), followed by Ag50-citrate, and lowest for Ag200-PVP. In vivo 10–50 µg Ag50-PVP triggered a dose-dependent pulmonary inflammatory milieu in NS and S mice, which was significantly higher in S mice and was dampened upon instillation of Ag200-PVP. Surprisingly, instillation of 1 µg Ag50-PVP significantly reduced OVA-induced inflammatory infiltrate in S mice and had no adverse effect in NS mice. Ag50-citrate showed similar beneficial effects at low concentrations and attenuated pro-inflammatory effects at high concentrations. The lung microbiome was altered by NPs instillation dependent on coating and/or mouse batch, showing the most pronounced effects upon instillation of 50 µg Ag50-citrate, which caused an increased abundance of operational taxonomic units assigned to Actinobacteria, Bacteroidetes, Firmicutes and Proteobacteria. However, no correlation with the biphasic effect of low and high Ag-NPs dose was found. Altogether, both in vitro and in vivo data on the pulmonary effects of Ag-NPs suggest the critical role of the size, dose and surface functionalization of Ag-NPs, especially in susceptible allergic individuals. From the perspective of occupational health, care should be taken by the production of Ag-NPs-containing consumer products.

## 1. Introduction

Nanoparticles (NPs) are structures which have a dimension in the range of 1–100 nm [1]. They are increasingly used in a wide field of applications, including medicine, cosmetics, food, health care, and for industrial purposes [2]. Among all nanoparticles in consumer products, silver is the most frequently used nanomaterial, because of its broad spectrum of properties, including antimicrobial effects against various pathogens such as bacteria, fungi, and viruses as well as anti-inflammatory activity [3]. However, the exposure to silver nanoparticles (Ag-NPs) can also be cytotoxic [4] and various properties of silver nanoparticles, such as size, distribution, coating, agglomeration, and dissolution rate, which meditate the advantages of nanotechnology, also influence their potential cytotoxicity [5,6,7]. Humans can be exposed to Ag-NPs by ingestion, dermal contact, or inhalation [8]. The latter is a major concern in occupational settings, for instance during the manufacture of nanoparticle-containing materials. Due to their small size and surface coating, e.g., quantum dot NPs applied to the lung may translocate and distribute systemically within hours [9]; particle size and the surface coating of silver nanoparticles are also key factors influencing cellular uptake and lung retention [5,10,11]. Interestingly, some studies of Ag-NPs have demonstrated cytoprotective activities, both in vitro and in vivo [12,13]. The existing data on the effects of Ag-NPs in pulmonary allergic inflammation are rather controversial. Antioxidant and anti-inflammatory effects of Ag-NPs have been shown in an ovalbumin-induced asthma mouse model [14] and this effect was shown to be dependent on the vascular endothelial growth factor (VEGF) signaling pathway and mucin regulation [15]. Immunomodulatory effects of Ag-NPs were also shown in a similar mouse rhinitis model [16]. On the contrary, pro-inflammatory and adjuvant effects have been described in a mouse asthma model by [16]. The authors investigated the inflammatory mechanism in vitro and conclude that Ag-NPs do not affect antigen uptake, but rather activate leucocytes and especially macrophages. Another mode of action of Ag-NPs might be triggered by the lung microbiome. The human lung microbiome harbors diverse microbial communities playing a critical role in immunomodulation as well as inflammation [17,18]. Although it has been proven in several studies that dietary nanoparticles severely influence the gut microflora [19,20], so far nothing is known about the effect of nanomaterials and their different physicochemical properties on the bacterial community composition of the lung; it’s likely that they affect the lung microbiome as well, with unknown consequences for human health.

The aim of this study was to investigate the effects of the dose, size and surface modification of silver NPs in vitro and in vivo, both in healthy mice and in a murine model of ovalbumin-specific allergic airway inflammation, including potential effects on the lung microbiome. We show that the dose of Ag-NPs is crucial for determining its inflammatory effects in vitro and its beneficial or pro-inflammatory and adjuvant effects in a mouse allergy model. Both in vitro and in vivo we show that the size plays an important role, showing that bigger particles are more inert. 

## 2. Results

### 2.1. Ag-NPs Exert Dose-Dependent Inflammatory Effects In Vitro

**Figure 1:**
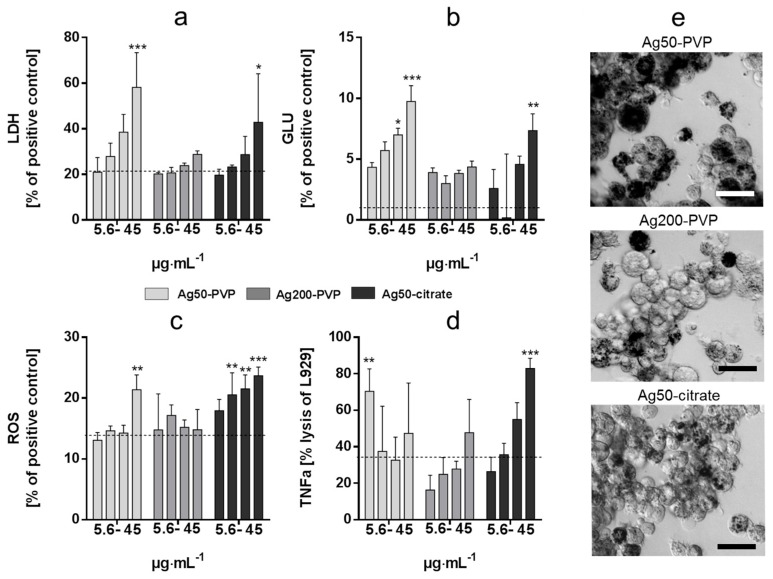
In vitro effects of Ag50-polyvinilpyrrolidone (PVP), Ag200-PVP and Ag50-citrate nanoparticles (NPs) on alveolar macrophages (NR8383). Four concentrations (5.6, 11.25, 22.5 and 45 µg·mL^−1^) were applied for 16 h (**a**,**b**,**d**) or 90 min (**c**). Supernatants were tested for activity of lactate dehydrogenase (LDH) (**a**) and glucuronidase (GLU) (**b**) relative to the positive control (Triton X-100). Release of H_2_O_2_ (**c**) is expressed relative to the positive control (zymosan). Bioactive tumor necrosis factor (TNF)-α was measured as percent lysis of L-929 reporter cells (**d**). Data are means±standard deviation (SD) from three independent experiments; dashed lines indicate mean values of untreated control cells; * *p* < 0.05, ** *p* < 0.01, *** *p* < 0.001. Uptake of silver NP (45 µg/mL, 16 h) is shown in (**e**). Note that while the bottom of the culture dish is clear, Ag-NPs appear as black grains localized intracellularly; bars: 20 µm.

All types of Ag-NPs were taken up by alveolar macrophages where they were visible as black agglomerates inside phagosomes. Up to the maximum concentration of 45 µg/mL, cells had cleared particles from the bottom of the culture vessel (Figure 1e). Cytotoxicity (lactate dehydrogenase (LDH) release) and induction of glucuronidase was maximal for Ag50-PVP, followed by Ag50 citrate and Ag200 PVP (Figure 1a,b) and significant effects commenced upon 22.5–45 µg Ag50-PVP per mL. The cytotoxicity of Ag200-PVP, albeit slightly dose-dependent, was not significantly different from control. Induction of biologically active TNF-α was dose dependent for Ag200-PVP and Ag50-citrate, but somewhat heterogeneous for Ag50-PVP, which was most potent at the lowest concentration (Figure 1d). With respect to the release of H_2_O_2_, Ag50-citrate was the most potent silver NP followed by Ag50 PVP, whereas Ag200-PVP elicited no effects (Figure 1c).

### 2.2. Ag50-PVP NPs Exert Dose-Dependent Effects In Vivo

**Figure 2:**
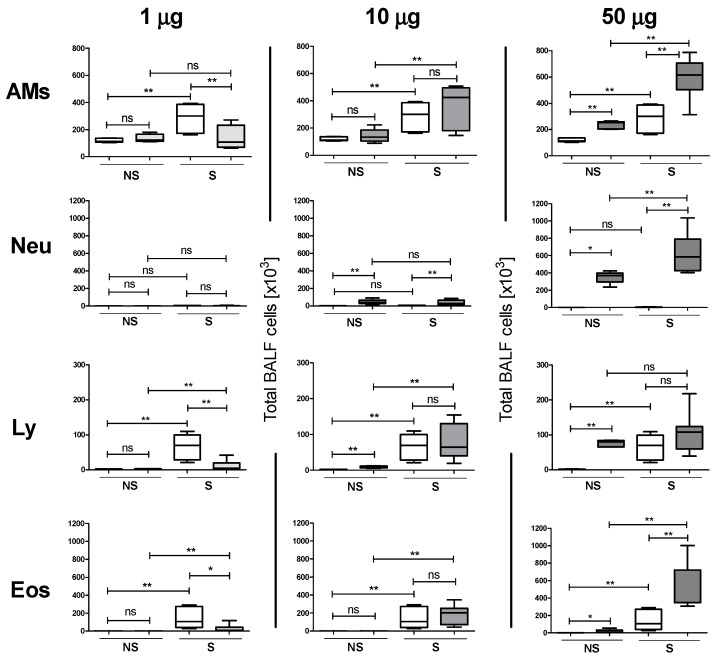
Ag50-PVP NPs exert dose-dependent effects in vivo. Bronchoalveolar lavage fluid *(*BALF) cell analysis. In each graph, on the left are non-sensitized mice (NS) and on the right sensitized (S) mice. White plots represent particle-free supernatant (SUP) controls and filled plots depict NPs-treated lungs. The dose is given above. AMs, alveolar macrophages; Neu, neutrophils; Ly, lymphocytes and Eos, eosinophils. (*n* = 5–8/group) * *p* < 0.05, ** *p* < 0.01; ns, not significant.

**Figure 3:**
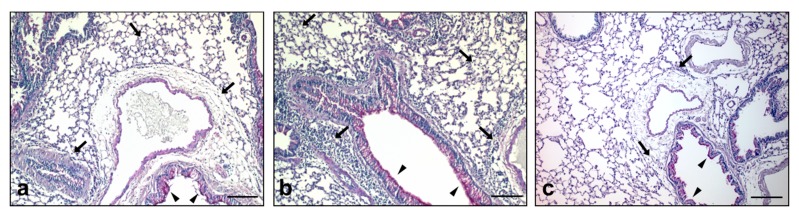
Ag50-PVP NPs exert dose-dependent effects in vivo*.* Histological analysis of representative lung sections of S mice 5 days after SUP (**a**), 50 μg Ag50-PVP (**b**) or 1 μg Ag50-PVP (**c**) and ovalbumin (OVA) challenge. Periodic acid Schiff (PAS) staining: inflammatory infiltrate (arrows) and mucus hypersecretion (arrowheads). Scale bar: 100 μm.

In order to investigate the effects of silver NPs in healthy and allergic mice, increasing concentrations of Ag**-**NPs (0, 1, 10 and 50 μg/mouse) were instilled intratracheally (i.t.) in the lungs of NS and S mice prior to OVA challenge. Broncho-alveolar lavage fluid (BALF) was analyzed for differential cell counts five days after OVA challenge. S mice instilled with particle-free supernatant (SUP, see methods section) showed increased macrophages, lymphocytes and eosinophils in the BALF compared to NS mice instilled with SUP. Instillation of 10 μg NPs induced only a mild increase in neutrophils both in S and in NS mice and of lymphocytes in NS mice only. The effects of the instillation were significantly higher in S mice compared to NS (Figure 2, center). Increasing the NPs dose to 50 μg led to a significant increase of the BALF cellular infiltrate compared to the respective SUPs, apart from lymphocytes in S mice, where the increase did not reach statistical significance. The increase in all BALF cells, apart again from lymphocytes, was significantly higher in S compared to NS mice (Figure 2, right). Surprisingly, in S mice the lowest NPs concentration used (1 μg) evoked a significant reduction of the inflammatory cell infiltrate in BALF, compared to the respective SUPs. The reduction was so strong that S mice showed a significant, although very scarce augmentation of lymphocytes and eosinophils compared to NS mice, whereas no effects were detected by the low dose Ag**-**NPs (Figure 2, left). These results were confirmed by a histological analysis of the lungs showing increased perivascular and peribronchiolar inflammatory infiltrate and mucus hypersecretion in S mice exposed to 50 μg Ag50 PVP prior to OVA challenge (Figure 3b), compared to S mice exposed to SUP prior to OVA challenge (Figure 3a). These pathological changes were significantly reduced in the lungs of S mice exposed to 1 μg Ag50 PVP prior to OVA challenge (Figure 3c). These results suggest a biphasic effect exerted by the dose of Ag-NPs applied to S mice: while high doses are strongly pro-inflammatory and adjuvant for allergic airway inflammation, low doses are immuno-modulatory and attenuate increments of macrophages, lymphocytes and eosinophils. 

#### 2.2.1. Ag-NPs Size and Surface Modification Significantly Affects Their In Vivo Response 

**Figure 4:**
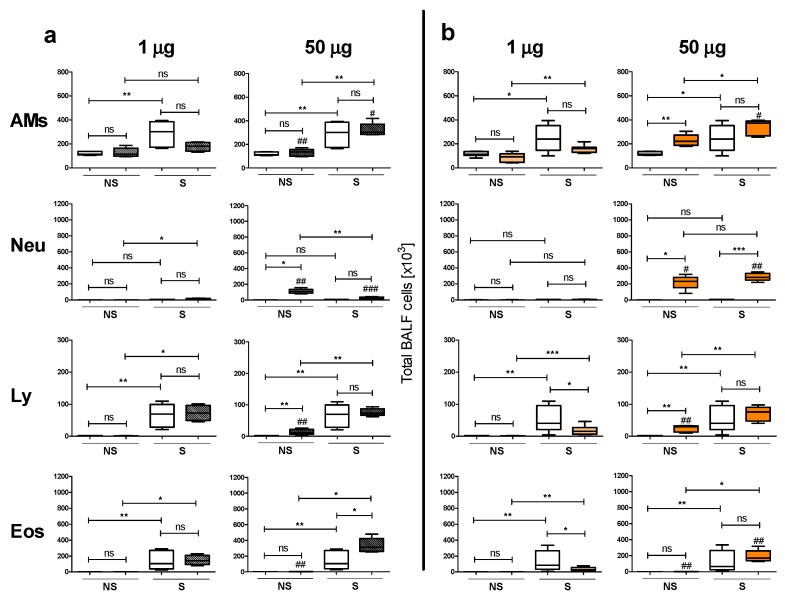
Ag-NPs size and surface modification significantly affects their in vivo response. BALF cell analysis following intratracheal (i.t.) instillation of (**a**) Ag200-PVP and (**b**) Ag50-citrate prior to OVA challenge. In each graph, on the left are non-sensitized mice (NS) and on the right sensitized (S) mice. White plots represent particle-free supernatant (SUP) controls and filled/colored plots depict NP-treated lungs. The dose is given above. AMs, alveolar macrophages; Neu, neutrophils; Ly, lymphocytes and Eos, eosinophils. (*n* = 5–10/group) * *p* < 0.05, ** *p* < 0.01; ns, not significant; ^#^ *p* < 0.05, ^##^ *p* < 0.01, ^###^ *p* < 0.001 vs. respective groups treated with Ag50-PVP.

Since i.t. instillation of 10 μg Ag50-PVP had the least effects on BALF cell analysis, we chose to proceed with the study of Ag200-PVP and Ag50-citrate using the doses of 1 and 50 μg NPs only. Again, S mice instilled with SUP showed increased macrophages, lymphocytes and eosinophils in the BALF compared to NS mice instilled with SUP (Figure 4). The instillation of the high dose of Ag200-PVP evoked a low, but significant, neutrophil and lymphocyte recruitment in NS animals and a low, but significant, increase in eosinophils in S animals, compared to SUP. The low dose had no effect compared to SUP. The effects of 50 μg Ag200-PVP in S animals were significantly higher compared to NS animals. The differences shown for 1 μg Ag200-PVP between S and NS animals are due to sensitization, rather than to NPs exposure. Analyzing the effects of Ag200-PVP vs. Ag50-PVP, we observed a high-dose-induced significant reduction in macrophages and neutrophils both in S and NS mice using the bigger particles; for lymphocytes and eosinophils the reduction did not reach statistical significance in S mice, only in NS mice (Figure 4a). Ag50-citrate at a high dose (50 μg) induced increased alveolar macrophages, neutrophils and lymphocytes in BALF compared to SUP in NS mice and increased neutrophils compared to SUP in S mice. The effects on S mice, apart from the infiltration of neutrophils, were significantly higher compared to NS mice. Most importantly, instillations of 1 μg Ag50-citrate had no effects on NS mice, but induced a significant reduction in lymphocytes and eosinophils in S mice compared to SUP, a key effect already observed with Ag50-PVP. Although at a low degree, the low-dose NP-induced inflammatory cell infiltration was higher in S mice compared to NS mice. Interestingly, by comparing the effects of 50 μg Ag50-citrate vs. 50 μg Ag50-PVP, we observed a significant reduction of macrophages, neutrophils and eosinophils in S mice and of neutrophils, lymphocytes and eosinophils in NS mice by using citrate-coated NPs. In the low-dose groups, no difference to Ag50-PVP was detected (Figure 4b). Taken together, the data show that Ag200-PVP induced less inflammatory response compared to the smaller counterpart and that Ag50-citrate induced a less robust inflammatory infiltrate compared to Ag50-PVP, maintaining their immuno-modulatory capabilities. 

#### 2.2.2. Ag-NPs Size and Surface Modification Significantly Affects BALF Total Protein and Cytokines and the Expression of Inflammatory Genes

**Figure 5:**
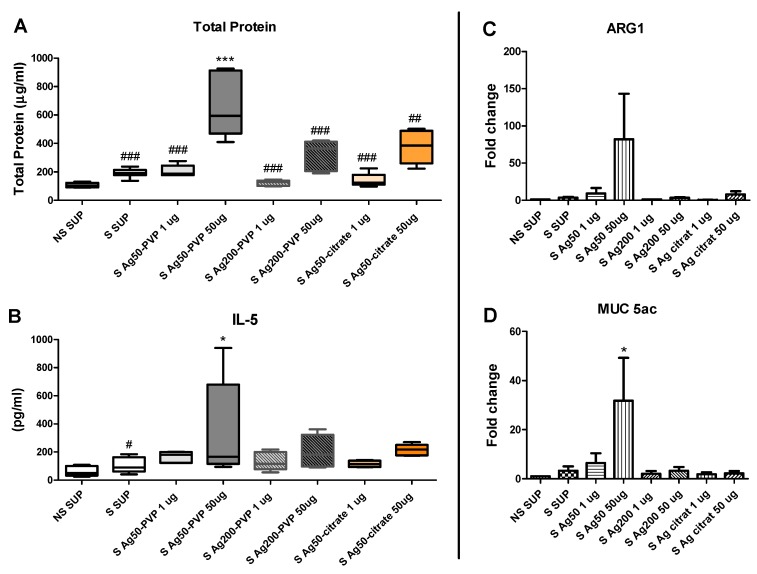
Ag-NPs size and surface modification significantly affects BALF total protein and cytokines and the expression of inflammatory genes. Total protein (**A**) and IL-5 (**B**) were analyzed in BALF (*n* = 5–6/group) and the expression of Arg1 (**C**) and Muc5ac (**D**) was analyzed in lung homogenates (*n* = 3–4/group); lungs were retrieved on day 53, 24 h after NPs or particle-free supernatant (SUP) instillation and OVA challenge. The groups are depicted underneath. White plots represent SUP controls and filled/colored plots represent NP-treated groups. * *p* < 0.05, *** *p* < 0.001 vs. NS SUP; ^#^ *p* < 0.05, ^##^ *p* < 0.01, ^###^ *p* < 0.001 vs. 50 μg Ag50-PVP.

Total protein and the Th1/Th2 cytokines interleukin (IL)-5, IL-13 and interferon (IFN)-γ were tested in the BALF of S mice one day after particle instillation and OVA challenge. The total protein of S mice exposed to 50 μg Ag50-PVP prior to OVA challenge was significantly increased compared to NS SUP. In all other groups tested, protein concentration was significantly lower compared to 50 μg Ag50-PVP (Figure 5a). Comparable results were obtained by evaluating IL-5 and IL-13 in BALF (IL-5 as an example, Figure 5b). On the contrary, no significant difference of IFN-γ in BALF was detected within the groups (data not shown). In addition, the expression of arginase (Arg)1, KC, IL-13 and mucin (Muc)-5ac was tested in lung homogenates. The expression of these genes showed a similar trend to total protein and Th2 cytokines, although only in MUC 5ac did the increase of 50 μg Ag50-PVP compared to NS SUP reach statistical significance (Figure 5d). In all other genes, we observed the same tendency, but no significant difference (Arg1 shown in Figure 5c, all other not shown).

### 2.3. Microbiome Analysis

To investigate if the pro- and anti-inflammatory effects of different doses of AgNPs and the effect of the coating of AgNPs with citrate were linked to the antibacterial properties of silver NPs [21], we performed an analysis of the lung microbiome using whole lung retrieved five days after instillation of 1 or 50 µg Ag50-PVP or Ag50-citrate (or respective supernatants) and OVA challenge. For technical reasons, Ag50-PVP and Ag50-citrate had to be analyzed in two separate mice batches, thus the coating effect might be confounded by batch effects. In total, 2,503,569 bacterial raw sequence reads were generated from the polymerase chain reaction (PCR) amplicons obtained in lung samples by Illumina sequencing. After noise filtering, chimera check and removing erroneous reads, 1,542,206 high quality partial 16S ribosomal ribonucleic acid (rRNA) gene sequences covering V1–V2 hypervariable regions with a minimum of 300 bp remained, which could be assigned to 7013 operational taxonomic units (OTUs) at 97% similarity. OTUs occurring in extraction blank and PCR negative controls were removed from the lung sample dataset. This did not influence the further data analysis as those accounted only for a minor fraction of the OTUs in the lung samples (Figure S1). To compare samples without statistical bias, 7144 reads were subsampled in all samples, reflecting the lowest observed read number (1 µg Ag50-PVP, replicate five). Rarefaction curves indicated that this sampling depth was sufficient for further analysis of samples at an OTU_97_ level (Figure S2).

Overall, bacterial richness ranged from 168 to 1048 OTUs and was significantly higher in Ag50-PVP/batch-1 samples (593–708 OTUs) compared to Ag50-citrate/batch-2 samples (168–697 OTUs) (Figure S2). No dose-dependent effect of Ag-NPs was observed on richness.

**Figure 6:**
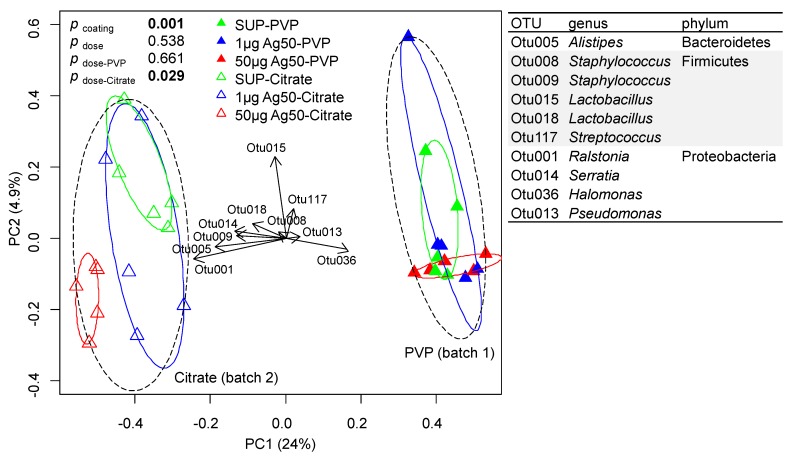
Lung microbiome beta: diversity analysis. Principal Component Analysis (PCA) plot generated from partial 16S ribosomal ribonucleic acid (rRNA) gene sequences clustered in operational taxonomic units (OTUs) with a 97% similarity level obtained from lung samples of two mice batches treated with either Ag50-PVP (batch 1) or Ag50-citrate (batch 2) NPs in different doses (1 µg, 50 µg) and the respective controls (SUP). The 10 most abundant OTUs are shown. Significant differences between the factors: coating/batch (Ag50-PVP/batch-1 vs. Ag50-citrate/batch-2) and dose (SUP, 1 µg, 50 µg), are indicated by *p* values < 0.05 (bold letters).

In total, 36 phyla were detected, with 69–92% of the annotated reads belonging to four major phyla: Actinobacteria, Bacteroidetes, Firmicutes and Proteobacteria (Figure S3). Those were ranked Proteobacteria > Firmicutes ≥ Bacteroidetes > Actinobacteria for citrate/batch-2 samples and Proteobacteria > Actinobacteria >> Firmicutes ≥ Bacteroidetes in Ag50-PVP/batch-1 samples. Additionally, Acidobacteria and Chloroflexi comprised up to 25% of reads in the Ag50-PVP/batch-1 samples but only up to 7% in the Ag50-citrate/batch-2 samples. The bacterial community composition of the lung microbiome was highly influenced by NPs-coating (PVP vs. citrate) and/or batch of test animals (Figure 6). A significant high-dose-effect could be observed for the Ag50-citrate-treated lung samples (batch 2) only, with increased abundance of Actinobacteria (*Aeromicrobium*), Bacteroidetes (*Prevotella, Alistipes*), Firmicutes (*Lactobacillales*_u, *Lachnospiraceae*_NK4A136_group, *Lachnospiraceae*_u, *Ruminococcaceae*_u) and Proteobacteria (*Ralstonia, Pantoae, Serratia*) in samples treated with 50 µg Ag-citrate compared to 1 µg Ag50-citrate and/or SUP samples. The relative abundance of *Acinetobacter* (Proteobacteria) was increased in the 1 µg Ag50-citrate samples compared to SUP samples, whereas *Schlegelella* (Proteobacteria) was affected in both Ag-NPs-treated samples compared to SUP (Table 1).

## 3. Discussion

Silver NPs, because of their intrinsic antimicrobial and therapeutic properties, are among the most commonly used engineered nanomaterials, appearing in dozens of commercial products [3,22]. However, their potential adverse effects have to be considered with attention. In our study we focused on assessing the dose-, size- and surface modification-dependence of the toxicity of silver NPs, utilizing materials with the same chemical composition, synthesized by the same method, but with different size and surface modification with citrate.

For the in vitro study we used doses ranging from 5.6 to 45 μg/mL for Ag50-PVP, Ag200-PVP and Ag50-citrate. The results show dose-dependent toxic effects of Ag-NPs, which are highest for Ag50-PVP as measured by LDH and glucuronidase activity, compared to the other two NPs tested; the release of ROS measured 90 min after NP exposure was significantly increased only at the highest concentrations of Ag50-PVP. The measurements of bioactive TNF-α show significant and the highest results compared to the other nanoparticles only at the lowest concentration, whereas increased Ag50-PVP failed to further increase bioactive TNF-α, probably due to the comparatively high cytotoxicity of Ag-50-PVP. This, along with the dose-dependency of the toxicological effect of Ag50-PVP, is in perfect accordance with the in vivo data. Here we set the highest amount of instilled AgNPs at 50 μg using a two-step calculation derived from our in vitro data. Given the largely complete uptake of sedimented NPs (see Figure 1e), we first calculated the mean macrophage AgNPs load at the cytotoxic concentration of 45 µg/mL, which resulted in a mean of 30 pg AgNPs/macrophage. Then, taking into account the number of macrophages per rat lung [23], we ended up with a dose of 30–60 µg NPs/mouse lung, which was expected to induce inflammatory effects in healthy animals. The dose was subsequently reduced to obtain a dose-response in diseased animals. Here we show the pro-inflammatory and adjuvant effects of Ag50-PVP starting at 10 μg (significant increase in BALF neutrophils) and reaching the highest level at 50 μg per lung, which was the highest dose tested. Ag50-PVP-induced BALF cell infiltration was significantly augmented in NS mice, in accordance with previous studies in rats [24], and was further amplified in S mice (Figure 2). This is in line with the concept that lung allergic inflammation is a susceptibility factor for the effects of particle exposure, where alterations in particle deposition and particle-induced oxidative stress were shown to play a critical role [25,26,27,28,29]. The five day time-point for BALF analysis was chosen according to previous data which indicated that this time-point was the most representative for the elicitation phase of the allergic response in our model [26,27]. Surprisingly, the instillation of only 1 μg Ag50-PVP prior to OVA challenge was shown to be immunomodulatory, as reported by the reduction (i) of inflammatory cells in BALF (Figure 2, left), (ii) of peribronchiolar, perivascular inflammation and (iii) of goblet cell hyperplasia in the lung sections (Figure 3c). The immunomodulatory effects of silver NPs have been described before as being due to an AgNPs-induced reduction of intracellular ROS generation, suppression of VEGF signaling and mucin regulation in allergic airway inflammation [14,15,16]. The novelty of our results consists in the biphasic dose-dependency of the effects of silver nanoparticles, as the same NPs displayed anti-inflammatory effects at low doses but pro-inflammatory effects at high doses. 

In perfect accordance with this, our in vitro and our in vivo data showed a significantly lower inflammatory potential of Ag200-PVP compared to Ag50-PVP, at the highest NPs concentration. The only effects on BALF cells upon instillation of 50 μg Ag200-PVP was a small increase in eosinophils in S mice and a slight increase in neutrophils and lymphocytes in NS mice. 

The comparison of the in vitro and in vivo data for Ag50-PVP and Ag50-citrate coating appears, however, not conclusive in all points. Our in vitro data for Ag50-citrate showed that its cytotoxic potential, reflected by the release of LDH and glucuronidase from alveolar macrophages, was lower for Ag50-citrate compared to Ag50-PVP. This was in line with in vivo findings for the high dose (50 µg/lung) of Ag50-citrate, which displayed a significantly lower pro-inflammatory potential compared to Ag50-PVP, both in NS and in S mice (Figure 4b, right), while there were no obvious differences in the immunomodulatory effects at the low-dose of Ag50-PVP or Ag50-citrate (Figure 4b, left). However, the dose-dependent increases in bioactive TNF-α and ROS production were gradually more pronounced for Ag50-citrate compared to Ag50-PVP (Figure 1), which inversely reflected the rank order observed in vivo. At present we can only speculate on possible reasons. It is known that silver nanoparticle preparations can shed a considerable amount of silver ions into the suspension, which are responsible for in vitro toxicity [30]. While instillation delivers particles as well as ions into the lung at the same time, the uptake of silver nanoparticles by alveolar macrophages in vitro relies on preceding gravitational settling, whereas the access of ions is diffusion limited. It appears possible that the Ag50-citrate preparation for in vitro studies had contained a lower amount of free ions (due to a reduction of silver ions by citrate), thus inferring less cytotoxicity, while particles elicited more TNF-α and ROS in less damaged cells. As ions and particles are delivered to the lung parenchyma simultaneously, in vivo results may deviate from in vitro studies. 

The results of the BALF cell analysis following instillations of Ag-NPs were supported by measurements of total protein and Th2 cytokines, and by analysis of the expression of lung inflammatory genes, like Arg1, a marker for alternatively-activated or type-2 macrophages, Muc5ac, linked to mucus hypersecretion and KC, responsible for the chemoattraction of granulocytes, following Ag50-PVP, Ag200-PVP and Ag50-citrate instillation in S animals (Figure 5). All these data show increased total protein and expression/release of inflammatory cytokines only in S mice following 50 μg Ag50-PVP prior to OVA challenge, and confirmed the reduced pro-inflammatory effects due to increased particle size and citrate coating. 

The effects of the Ag-NPs size and coating on pulmonary inflammation have been extensively studied in different rodent models and strains, with different particle sizes and concentrations [10,31,32,33,34,35]. Little difference has been shown following instillations of Ag-NPs coated with citrate or PVP, although citrate appears to increase the short-term lung inflammatory infiltrate and to slightly influence the long-term lung burden of Ag-NPs. Obviously, differences in particle chemical composition and experimental model used can account for discrepancies in the results. In contrast, there is a common agreement that smaller Ag-NPs are more toxic than bigger particles. This effect is multi-causal. First, smaller nanoparticles have been shown to cross cellular barriers more easily by specific uptake and endocytic processes than bigger particles, enhancing their translocation to the interstitium [36]; on the contrary, bigger particles undergo a more rapid clearance from large airways compared to smaller ones [10]; furthermore, smaller NPs release greater amounts of toxic silver ions compared to larger Ag-NPs, proportionally to the greater surface area/μg of deposited Ag-NPs [7,31]; finally, smaller Ag-NPs create a greater silver burden and increase their persistence in alveolar macrophages [37]. This last study is corroborated by our in vitro observation on particle uptake, which shows more uptake of Ag50-PVP by alveolar macrophages compared to Ag200-PVP and to Ag50-citrate. 

The lungs of humans harbor diverse microbial communities which play an important role in immunomodulation and inflammation [17,18] and, as shown here, might be influenced by NPs instillation, too. Thus, we sought to investigate the effects of the dose and coating of AgNPs on the lung microbiome. Interestingly, we observed neither a significant effect of 1 µg, nor of 50 µg Ag50-PVP on the lung microbiome, although in vivo data indicated strong anti- and pro-inflammatory capabilities of these treatments, respectively. It may therefore be concluded that the biphasic immuno-modulatory effect of Ag50-PVP is not due to silver-NPs-induced changes of the lung microbiome. The general lack of effect of Ag50-PVP is also in contrast to a study investigating the influence of dietary PVP-coated silver nanoparticles on gut microbiota in mice [20], showing a dose-dependent effect on bacterial evenness and microbial community structure. However, the main responding taxa in this study belonged to Firmicutes and Bacteroidetes, which dominate the gut microbiome (>95%) while in our study those phyla accounted only for 2–38% of all reads. Although not significant, Gammaproteobacteria were highest after 50 µg Ag50-PVP treatment. This is in accordance with results obtained from the gut microbiome showing correlations between inflammation and Gammaproteobactera blooms (reviewed in [38]). 

In contrast to PVP-coating, silver NPs coated with citrate affected the bacterial community in mouse lungs, most pronounced after the 50 μg dose. While only two OTUs (Proteobacteria) were increased for the lower NPs dose compared to the control treatment, an additional nine OTUs were increased for the higher NPs dose, belonging to Actinobacteria, Bacteroidetes, Firmicutes and Proteobacteria. This is in accordance with the results from the gut microbiome, where Lachnospiraceae (Firmicutes) and Rikenellaceae (Bacteroidetes) increased with increasing silver NPs concentrations [20]. 

As the effects on both the lung and gut microbiome were seen for citrate but not for PVP coating, the type of NPs coating seems to be a dominant factor in shaping the silver-sensitive lung microbiome. However, we have to consider that, due to technical reasons, two separate batches of mice were used for the PVP- and citrate-coated NPs instillations and it is known that the housing conditions of animals may significantly influence the lung microbiome [39]. Consequently, we cannot yet distinguish between the effect of coating and the effect of batch and/or cage on the lung bacterial community composition. Further studies should address how the lung microbiome can be influenced by both, coating and dose of AgNPs, and vice-versa, if and to which extent alterations in the lung microbiome can trigger the observed biphasic immuno-modulatory effect of AgNPs. 

Altogether, both in vitro and in vivo data on the pulmonary effects of Ag-NPs suggest a critical role for the size, dose and coating of Ag-NPs, especially when exposures occur in susceptible allergic individuals. From the perspective of occupational health, care should be taken in the production of Ag-NPs-containing consumer products. Workplace exposure and health hazard assessment studies have reported that concentrations of AgNPs in the processes of manufacturing and integration of AgNPs into various products can reach up to 1.35 μg/m^3^ [40,41]. Derived dosimetric evaluations have identified the liver as the primary and most sensitive target organ, followed by the lung, due to particle inhalation. The derived dosimetrically-adjusted benchmark dose causing lung inflammation in the workplace is considered to be between 1 and 20 μg/lung [42]. There are no reports on exposure to silver NPs at the workplace in susceptible individuals, as in people affected by lung disease like asthma, but considering the strong dose-dependent immunomodulating effects observed by pulmonary exposure to Ag-NPs in sensitized mice, we might consider special protection for asthmatics who are exposed to NPs in the work place.

## 4. Materials and Methods

### 4.1. Preparation of Silver Nanoparticle Dispersions

Ag50-PVP, Ag200-PVP and Ag50-citrate NPs were obtained from Bayer Technology Services GmbH (Leverkusen, Germany), as aqueous stock suspensions (20% *w*/*w*), and prepared and characterized as described in [43]. For routine testing with the macrophage model [44], NPs were diluted in cell culture medium (F-12K; Biochrom GmbH, Berlin, Germany) or glucose-containing phosphate-buffered Krebs Ringer (KRPG) to a working concentration of 180 µg/mL by brief ultrasonication (10 s), using a 3 mm probe adjusted to 50 W (Vibra Cell™, Sonics & Materials, Danbury, CT, USA). Further dilutions were made with the same medium as outlined in the text. 

For in vivo NPs testing, all Ag-NPs were freshly diluted in *aqua ad iniectabilia* (Braun Melsungen AG, Puchheim, Germany) and buffered with PBS to physiological pH to the final concentration of 1 mg/mL straight before intratracheal instillation. Supernatant controls (SUP) were obtained by hard sedimentation (100,000 g, 15 h). 

Additional NPs characteristics can be found in Table 2.

### 4.2. In Vitro Toxicity Testing

Rat alveolar macrophage cell line NR8383 was cultured in 175 cm^2^ culture flasks with 50 mL F12-K medium supplemented with 2 mM glutamine, penicillin, and 15% heat inactivated fetal calf serum (FCS) at 37 °C/5% CO_2_ purchased from PAA Laboratories GmbH (Cölbe, Germany). In vitro toxicity testing was carried out with NR8383 as described in [44]. In brief, cells were harvested, adjusted to 1.5 × 10^6^ live cells/mL, seeded at a density of 3 × 10^5^ cells per well into 96-well plates, and incubated over night with reduced serum concentration (5%) under cell culture conditions (37 °C, 5% CO_2_). On the next day the plates were used either for the ROS formation assay or cytotoxicity testing: For cytotoxicity testing the supernatants were replaced by particle containing suspension which was prepared in serum-free F-12K and incubated for 16h. Supernatants were centrifuged at 200× *g* for 10 min and 50 µL from each well was analyzed by colorimetric tests for LDH activity (Roche Cytotoxicity Kit; Roche Dignostic GmbH, Penzberg, Germany), and glucuronidase activity using 0.2 M sodium acetate buffer (pH 5) with 13.3 mM p-nitrophenyl-d-glucuronide (Sigma-Aldrich Chemie GmbH, Taufkirchen, Germany) and 0.1% Triton X-100. Bioactive TNF-α was measured by applying 50 µL of the supernatant to a confluent layer of L-929 fibroblasts in the presence of actinomycin D. TNF-α activity was expressed as percent killing activity of the crystal violet stained cells. All colorimetric measurements were carried out with a Tecan 200Pro plate reader (Tecan, Germany). 

To detect extracellular H_2_O_2_/ROS formation, supernatants were replaced by particle suspensions prepared in KRPG buffer (Krebs-Ringer phosphate glucose buffer). A reaction mix containing horseradish peroxidase and AmplexRed^®^ was added for 90 min after which the increase in resorufin was detected colorimetrically at 450 nm. Measurements were corrected for cell-free particle controls, and expressed as percent of the positive zymosan control (180 µg/mL).

### 4.3. In Vivo Ag-NP Testing

#### 4.3.1. Animals and Study Design

**Figure 7:**
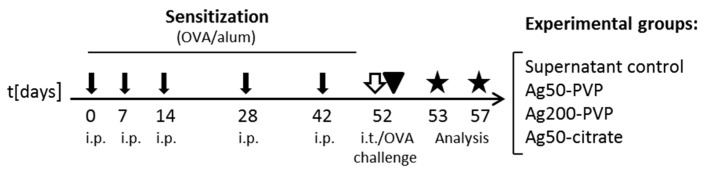
Experimental protocol. Sensitized (S) mice were intraperitoneally injected with 1 µg OVA in phosphate-buffered saline (PBS)/alum (black arrows). Non-sensitized (NS) mice were injected with PBS/alum. On day 52, mice were intratracheally instilled with Ag-NPs or with SUP (white arrow) and subsequently challenged with OVA aerosol (arrowhead). Mice were sacrificed on day 53 or on day 57 (stars).

Female, 6–10 weeks old BALB/c mice were obtained from Charles River (Sulzfeld, Germany), housed under specific pathogen free conditions in individually ventilated cages (VentiRack, Biozone, Margate, UK) and fed a standard diet and water ad libitum. The study was conducted under federal guidelines for the use and cares of laboratory animals and was approved by the Government of the District of Upper Bavaria (Approval No. 55.2-1-54-2532-156-12) and the Animal Care and Use Committee of the Helmholtz Center Munich. To evaluate the adjuvant effect of nanoparticles, a protocol of mild allergic inflammation in the lung was used as previously described (Figure 7) [26]. Briefly, mice were sensitized by repetitive intraperitoneal (i.p.) injections of 1 µg OVA (grade VI; Sigma-Aldrich Chemie GmbH, Taufkirchen, Germany) in PBS adsorbed to Alum (2.5 mg; Thermo Scientific, Rockford, IL, USA) on days 0, 7, 14, 28 and 42. Blood samples were taken before and after sensitization. OVA/Alum sensitized (S) mice, compared to non-sensitized (NS) mice, were characterized by high titers of OVA-specific IgE (8.05 ± 1.64 vs. 0.1 ± 0.03 μg/mL). At day 52 mice were intratracheally (i.t.) instilled with Ag-NPs (1, 10 or 50 μg), or with the correspondent SUP, using a graduated syringe. NPs instillation was followed by OVA-aerosol challenge for 20 min with 1% ovalbumin in PBS delivered by a Pari-Boy nebulizer (Pari, Starnberg, Germany). Analysis of BALF total protein and inflammatory cytokines was performed on day 53; analysis of BALF inflammatory infiltrate, evaluation of lung histology and analysis of lung microbiome were performed on day 57. For technical reasons, Ag50-PVP and Ag50-citrate had to be analyzed in two separate mice batches.

#### 4.3.2. Bronchoalveolar Lavage, Real-Time PCR, Histology, Measurement of Total Protein and Cytokines

Bronchoalveolar lavage and measurement of the total protein and cytokines in BALF were performed as previously described in [26,28]. For the histology, after bronchoalveolar lavage (BAL), the lungs were excised and the left lobe fixed in 4% buffered formalin and embedded in paraffin. Sections of 3 µm thickness were stained with hematoxylin-eosin (H&E) and periodic acid Schiff (PAS). For the real-time PCR, total RNA was extracted from snap-frozen lung tissue as in [25]. A total of 0.4 μg of RNA was converted to cDNA following manufacturing protocol (Fermentas, ThermoFischer Scientific, Waltham, MA, USA). Real-time PCR was performed with SYBR Green and ViiA™ 7 thermocycler (Applied Biosystems, Foster City, CA, USA). Relative expression levels were calculated using the 2^ddct^ method, normalized to ACTB. The primer sequences were the following: Arg1 (forward) 5′-AGAGATTATCGGAGCGCCTT-3′ (reverse) 5′-TTTTTCCAGCAGACCAGCTT-3′; Muc5ac (forward) 5′-TGGAGTCAGCACGAAAACAG-3′ (reverse) 5′-GCACTGGGAAGTCAGTGTCA-3′; IL-13 (forward) 5′-TGTGTCTCTCCCTCTGACCC-3′ (reverse) 5′-CACACTCCATACCATGCTGC-3′; KC (forward) 5′-CCACACTCAAGAATGGTCGC-3′ (reverse) 5′-TCTCCGTTACTTGGGGACAC-3′; ACTB (forward) 5′-TTCTTTGCAGCTCCTTCGTT-3′ (reverse) 5′-ATGGAGGGGAATACAGCCC-3′. Data were considered significant with a *p* value ≤ 0.05 in a confidence interval of 95%.

#### 4.3.3. Lung Microbiome Analysis: DNA Extraction, Sequencing Procedure and Data Processing

DNA was extracted from the mouse lungs retrieved five days after NPs exposure and OVA challenge using the protocol for phenol-chloroform extraction from mouse tail biopsies described by the Jackson Laboratory (https://www.jax.org) with the following modifications: (i) the DNA digestion buffer consisted of 1× PBS buffer containing 0.5 mg mL^−1^ proteinase K (Thermo Fisher Scientific, Waltham, MA, USA); (ii) the phenol-chloroform-isoamyl alcohol (25:24:1) extraction step was performed twice; (iii) the centrifugation after the ethanol precipitation steps was performed at 4 °C for 10 min; and (iv) the DNA was resolved in nuclease-free water. Subsequently, microbial DNA was enriched using a NebNext Microbiome DNA Enrichment Kit (New England Biolabs, Ipswich, MA, USA) with 5 µg of input DNA, resulting in a final DNA amount from 0.2 to 1.3 µg.

Amplicon sequencing was performed on a MiSeq Illumina (Illumina, San Diego, CA, USA). The universal eubacterial primers 27f (5′-*TCGTCGGCAGCGTCAGATGTGTATAAGAGACAG*-AGAGTTTGATCMTGGC-3′) and 357r (5′-*GTCTCGTGGGCTCGGAGATGTGTATAAGAGACAG*-CTGCTGCCTYCCGTA-3′) covering the V1–V2 hypervariable regions of the 16S rRNA gene [45] were extended with sequencing adapters (in italics) to match the Illumina indexing primers. Each 25 μL PCR reaction was done in triplicate and contained 1× NebNext High Fidelity Mastermix (New England Biolabs), 0.5 mM of each primer and 40 ng of template DNA. Sequencing PCR was performed starting with 98 °C for 5 min, followed by 30 cycles of 98 °C for 10 s, 60 °C for 30 s, 72 °C for 30 s, and final extension at 72 °C for 5 min. The DNA extraction purity and PCR specificity were confirmed by a blank extraction without lung tissue and three PCR control samples without template DNA which were treated similarly. PCR products were visualized on 1% agarose gel to verify the product size. PCR triplicates were pooled and purified using AMPure beads XP (Beckman Coulter, Brea, CA, USA). After measuring the fragment size and concentration with a DNA 7500 Chip on a Bioanalyzer 2100 device (Agilent, Santa Clara, CA, USA), indexing PCR was performed as follows: 98 °C for 30 s, followed by eight cycles of 98 °C for 10 s, 55 °C for 30 s, 72 °C for 30 s, and final extension at 72 °C for 5 min. Each 25 μL PCR reaction contained 1× NebNext High Fidelity Mastermix (New England Biolabs), indexing primer 1 (N7xx) and indexing primer 2 (S5xx) and 10 ng of template DNA [46,47]. All samples were purified using AMPure beads XP (Beckman Coulter, Brea, CA, USA). Subsequently, PCR products were validated via Bioanalyzer 2100 using a DNA 7500 Chip (Agilent, Santa Clara, CA, USA) and quantified via Quant-iT Pico Green dsDNA Assay Kit (Thermo Fisher, Waltham, MA, USA). Libraries were pooled to a final concentration of 4 nM for the MiSeq sequencing run.

Raw sequences were processed with MOTHUR v.1.39.3 [48] as described previously in [46,47]. Briefly, contiguous sequences were formed by removing barcodes and primers. Sequences were checked for chimeras by alignment to the SILVA reference files provided by MOTHUR and were subsequently classified using the SILVA database (release 123). After removing mitochondrial sequences, a distance matrix from the high-quality aligned sequences was calculated. Using the furthest neighbor clustering algorithm at 97% sequence similarity, operational taxonomic units (OTUs) were received which were used for calculation of rarefaction curves. The nucleotide sequence data obtained in this study have been deposited in the NCBI Sequence Read Archive under study accession number SRP114665. 

#### 4.3.4. Statistical Analysis

In vitro data were generated in triplicate and at least three independent repetitions were carried out. Data were expressed as mean ± standard deviation (SD) and analyzed using Graph Pad Prism software (Version 6; GraphPad Software Inc., La Jolla, CA, USA). Values were compared to non-treated vehicle controls using 2-way analysis of variance (ANOVA) and Dunnett’s post hoc multiple comparison test. For the in vivo data, results are shown as boxplots indicating minimum, 25% percentile, median, 75% percentile and maximum, or as mean ± standard deviation (SD). Statistical significance among groups was determined by the Mann-Whitney *U* test (GraphPad Prism, La Jolla, CA, USA). Results were considered significant as * *p* < 0.05, ** *p* < 0.01, *** *p* < 0.001. Lung microbiome data were analyzed in R v3.4.0 (http://www.R-project.org/) using multivariate analysis of variance (Adonis function) based on Yue Clayton dissimilarity and Euclidean distances of Hellinger transformed data as described previously in [46,47]. Replicate similarity (*n* = 5) was checked by clustering all OTUs according to a dissimilarity matrix based on the Yue Clayton coefficient, resulting in the unweighted pair group method (UPGMA) dendrogram. The following R packages were used for calculation of significant differences and data visualization: agricolae v1.2-4, ape v4.1, ggplot2 v2.2.1, gplots v3.0.1, mass v7.3-47 and vegan v2.4-3.

## Figures and Tables

**Table 1:** Mean relative abundance (%) of OTUs that are different between the dose groups in Ag50-citrate-treated lung samples (*n* = 5).

OTU Number	1 µg	50 µg	SUP	Phylum	Class	Order	Family	Genus
Otu217	0.00	0.41	0.00	Actinobacteria	Actinobacteria	Propionibacteriales	Nocardioidaceae	*Aeromicrobium*
	b	a	b					
Otu172	0.00	0.40	0.08	Bacteroidetes	Bacteroidia	Bacteroidales	Prevotellaceae	*Prevotella*
	b	a	ab					
Otu005	3.66	7.02	2.23	Bacteroidetes	Bacteroidia	Bacteroidales	Rikenellaceae	*Alistipes*
	b	a	b					
Otu162	0.13	0.31	0.00	Bacteroidetes	Bacteroidia	Bacteroidales	Rikenellaceae	*Alistipes*
	ab	a	b					
Otu235	0.00	0.36	0.00	Firmicutes	Bacilli	Lactobacillales	unclassified	unclassified
	b	a	b					
Otu027	0.39	1.89	0.08	Firmicutes	Clostridia	Clostridiales	Lachnospiraceae	NK4A136_group
	b	a	b					
Otu102	0.05	0.61	0.13	Firmicutes	Clostridia	Clostridiales	Lachnospiraceae	unclassified
	b	a	b					
Otu156	0.06	0.34	0.12	Firmicutes	Clostridia	Clostridiales	Lachnospiraceae	unclassified
	b	a	ab					
Otu284	0.01	0.28	0.01	Firmicutes	Clostridia	Clostridiales	Ruminococcaceae	unclassified
	b	a	b					
Otu001	10.12	11.35	5.02	Proteobacteria	Beta-Proteo	Burkholderiales	Burkholderiaceae	*Ralstonia*
	ab	a	b					
Otu063	0.42	0.38	0.04	Proteobacteria	Beta-Proteo	Burkholderiales	Comamonadaceae	*Schlegelella*
	a	a	b					
Otu111	0.00	0.37	0.02	Proteobacteria	Gamma-Proteo	Enterobacteriales	Enterobacteriaceae	*Pantoea*
	b	a	ab					
Otu026	0.15	1.88	0.98	Proteobacteria	Gamma-Proteo	Enterobacteriales	Enterobacteriaceae	*Serratia*
	b	a	ab					
Otu046	0.80	0.36	0.18	Proteobacteria	Gamma-Proteo	Pseudomonadales	Moraxellaceae	*Acinetobacter*
	a	ab	b					

Differences (*p* < 0.05) between ad-hoc grouping (ANOVA) of the doses (indicated by: a, ab and b).

**Table 2:** Particle characterization.

Particle Property	Ag50-PVP	Ag200-PVP	Ag-Citrate
Primary particle size (TEM)	97 nm	134 nm	20 nm
Specific surface size (BET)	6.2 m^2^/g	4.5 m^2^/g	30 m^2^/g
Average size in H_2_O (AUC)	77 nm	95 nm	38 nm
crystallinity	cubic	cubic	cubic
Surface chemistry (XPS) in element %	C59, O18, Ag16, Na8	C77, O10, Ag12, Na1, N1	C21, O15, Ag62, Na2
Isoelectric point (pH)	3.6	4.2	2
Zeta potential at pH 7.4 (mV)	−7	−7	−45

TEM: transmission electron microscopy; BET: nitrogen adsorbing surface according to the Brunauer-Emmett-Teller equation; AUC: analytical ultracentrifugation; XPS: X-ray photoelectron spectroscopy [43].

## Supplementary Materials

The following are available online at http://www.mdpi.com/2079-4991/7/10/300/s1. Figure S1: Venn diagram of shared OTUs between lung samples (Ag50-PVP/batch-1, Ag50-citrate/batch-2), extraction blank (Blank) and PCR negative controls (Neg). Figure S2: Rarefaction curves of partial 16S rRNA gene sequences obtained from lung samples at 97% similarity level (subsampled to 7144 reads). Figure S3: Relative abundance of sequences assigned to phylum level in lung samples based on partial 16S rRNA gene sequences.
