# Supplementary material for: Pro-Inflammatory versus Immunomodulatory Effects of Silver Nanoparticles in the Lung: The Critical Role of Dose, Size and Surface Modification
Source: Nanomaterials (Basel). 2017 Sep 29;7(10):300. doi: 10.3390/nano7100300 (PMC5666465; doi:10.3390/nano7100300)
Supplement: Supplementary file 1 [file nanomaterials-07-00300-s001.pdf]

Supplementary Materials

Pro-inflammatory Versus Immunomodulatory Effects of Silver Nanoparticles in the Lung: the Critical Role of Dose, Size and Surface Modification

Francesca Alessandrini [1,*], Antje Vennemann [2], Silvia Gschwendtner [3], Avidan U. Neumann [4,5], Michael Rothballer [6], Tanja Seher [1], Maria Wimmer [1], Susanne Kublik [3], Claudia Traidl-Hoffmann [4,5], Michael Schloter [3], Martin Wiemann [2] and Carsten B. Schmidt-Weber [1]

1. Center of Allergy and Environment (ZAUM), Technical University and Helmholtz Center Munich, Member of the German Center for Lung Research (DZL), Ingolstädter Landstr. 1, 85764 Neuherberg, Germany; tanja.seher@helmholtz-muenchen.de (T.S.); maria.wimmer84@web.de (M.W.); csweber@tum.de (C.B.S.-W.)
2. IBE R & D Institute for Lung Health gGmbH, Mendelstr. 11, 48149 Münster, Germany; vennemann@ibe-ms.de (A.V.); martin.wiemann@ibe-ms.de (M.W.)
3. Research Unit for Comparative Microbiome Analysis, Helmholtz Center Munich, Ingolstädter Landstr. 1, 85764 Neuherberg, Germany; silvia.gschwendtner@helmholtz-muenchen.de (S.G.); susanne.kublik@helmholtz-muenchen.de (S.K.); schloter@helmholtz-muenchen.de (M.S.)
4. Chair and Institute of Environmental Medicine, UNIKA-T, Technical University of Munich and Helmholtz Center Munich, Neusäßer Str. 17, 86156 Augsburg, Germany
5. CK-CARE, Christine Kühne-Center for Allergy and Research and Education, Herman-Burchard-Strasse 1, 7265 Davos Wolfgang, Switzerland; auneumann@gmail.com (A.U.N.); claudia.traidl-hoffmann@tum.de (C.T.-H.)
6. Institute of Network Biology (INET), Helmholtz Center Munich, Ingolstädter Landstr. 1, 85764 Neuherberg, Germany; rothballer@helmholtz-muenchen.de (M.R.)

* Correspondence: franci@helmholtz-muenchen.de (F.A.); Tel.: +49-89-3187-2524 (F.A.)

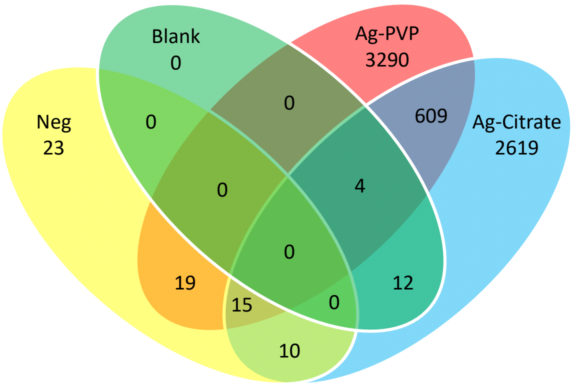

Figure S1.: Venn diagram of shared OTUs between lung samples (Ag50-PVP/batch-1, Ag50-citrate/batch-2), extraction blank (Blank) and PCR negative controls (Neg)

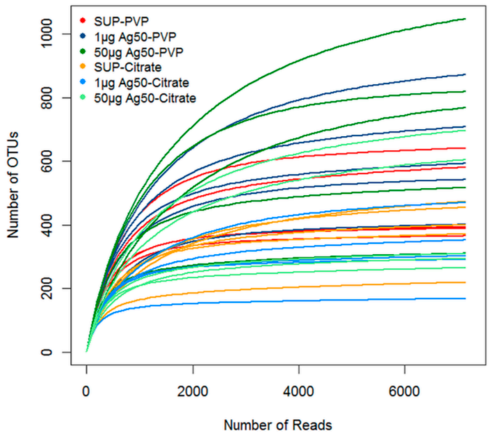

Figure S2. : Rarefaction curves of partial 16S rRNA gene sequences obtained from lung samples at 97% similarity level (subsampled to 7144 reads)

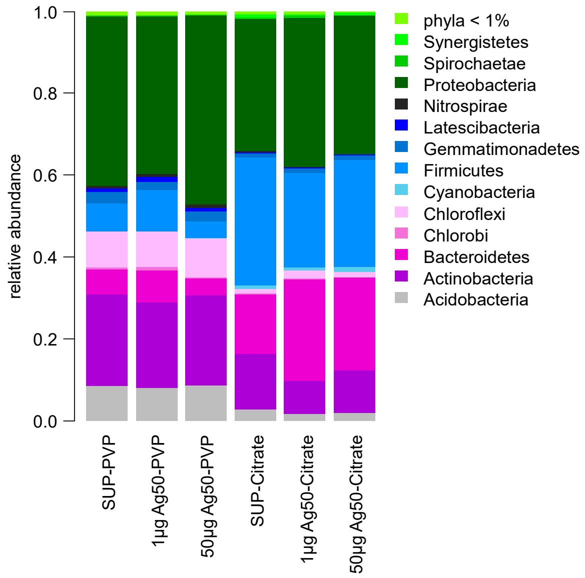

Figure S3. : Relative abundance of sequences assigned to phylum level in lung samples based on partial 16S rRNA gene sequences.
